# Mammillary body abnormalities and cognitive outcomes in children cooled for neonatal encephalopathy

**DOI:** 10.1111/dmcn.15453

**Published:** 2022-11-06

**Authors:** Arthur P. C. Spencer, Maarten H. Lequin, Linda S. de Vries, Jonathan C. W. Brooks, Sally Jary, James Tonks, Frances M. Cowan, Marianne Thoresen, Ela Chakkarapani

**Affiliations:** ^1^ Translational Health Sciences, Bristol Medical School University of Bristol Bristol UK; ^2^ Clinical Research and Imaging Centre University of Bristol Bristol UK; ^3^ Department of Radiology and Nuclear Medicine University Medical Center Utrecht/Wilhelmina Children's Hospital Utrecht the Netherlands; ^4^ Princess Máxima Center for Pediatric Oncology Utrecht the Netherlands; ^5^ Department of Neonatology University Medical Center Utrecht Utrecht the Netherlands; ^6^ Department of Neonatology Leiden University Medical Center Leiden the Netherlands; ^7^ School of Psychology University of East Anglia Norwich UK; ^8^ University of Exeter Medical School Exeter UK; ^9^ Department of Paediatrics Imperial College London London UK; ^10^ Faculty of Medicine Institute of Basic Medical Sciences, University of Oslo Oslo Norway; ^11^ Neonatal Intensive Care Unit St Michael's Hospital, University Hospitals Bristol and Weston NHS Foundation Trust Bristol UK

## Abstract

**Aim:**

To evaluate mammillary body abnormalities in school‐age children without cerebral palsy treated with therapeutic hypothermia for neonatal hypoxic–ischaemic encephalopathy (cases) and matched controls, and associations with cognitive outcome, hippocampal volume, and diffusivity in the mammillothalamic tract (MTT) and fornix.

**Method:**

Mammillary body abnormalities were scored from T1‐weighted magnetic resonance imaging (MRI) in 32 cases and 35 controls (median age [interquartile range] 7 years [6 years 7 months–7 years 7 months] and 7 years 4 months [6 years 7 months–7 years 7 months] respectively). Cognition was assessed using the Wechsler Intelligence Scale for Children, Fourth Edition. Hippocampal volume (normalized by total brain volume) was measured from T1‐weighted MRI. Radial diffusivity and fractional anisotropy were measured in the MTT and fornix, from diffusion‐weighted MRI using deterministic tractography.

**Results:**

More cases than controls had mammillary body abnormalities (34% vs 0%; *p* < 0.001). Cases with abnormal mammillary bodies had lower processing speed (*p* = 0.016) and full‐scale IQ (*p* = 0.028) than cases without abnormal mammillary bodies, and lower scores than controls in all cognitive domains (*p* < 0.05). Cases with abnormal mammillary bodies had smaller hippocampi (left *p* = 0.016; right *p* = 0.004) and increased radial diffusivity in the right MTT (*p* = 0.004) compared with cases without mammillary body abnormalities.

**Interpretation:**

Cooled children with mammillary body abnormalities at school‐age have reduced cognitive scores, smaller hippocampi, and altered MTT microstructure compared with those without mammillary body abnormalities, and matched controls.

**What this paper adds:**

- Cooled children are at higher risk of mammillary body abnormalities than controls.
- Abnormal mammillary bodies are associated with reduced cognitive scores and smaller hippocampi.
- Abnormal mammillary bodies are associated with altered mammillothalamic tract diffusivity.

## What this paper adds

We investigated mammillary body abnormalities in early school‐age children without cerebral palsy cooled for neonatal hypoxic‐ischaemic encephalopathy, and controls matched for age, sex, and socioeconomic status. A third of cooled children had abnormal mammillary bodies, compared to none of the control group. Cooled children with abnormal mammillary bodies had reduced cognitive outcome scores, smaller hippocampi, and altered diffusion properties in the mammillothalamic tract compared to cooled children without mammillary body abnormalities, and controls.

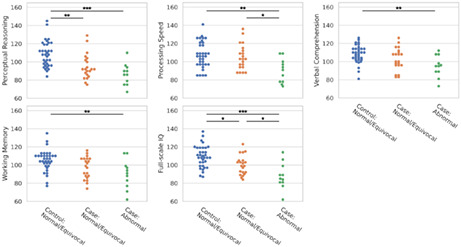

**Abbreviations**
DWI: diffusion‐weighted imaging
FOD: fibre orientation distribution
FSL: FMRIB software library
HIE: hypoxic–ischaemic encephalopathy
MTT: mammillothalamic tract
ROI: region of interest
TBV: total brain volume

Neonatal hypoxic–ischaemic encephalopathy (HIE) secondary to perinatal asphyxia leads to a high risk of death or disability, including cerebral palsy (CP).^1^ Therapeutic hypothermia reduces the risk of death and severe disability compared with normothermia management and is now standard care for HIE in high‐income countries. Despite the benefits of therapeutic hypothermia, some early school‐age children who underwent therapeutic hypothermia for HIE and did not develop CP had reduced cognitive, motor, and behavioural scores^3^, ^4^ and structural brain alterations^5^, ^6^, ^7^ compared with typically developing controls.

The mammillary bodies are known to be important for memory function.^8^ Mammillary bodies receive inputs from the hippocampi via the fornix, and project to the anterior thalamic nuclei via the mammillothalamic tract (MTT); these connections form part of the Papez circuit.^8^, ^9^ Damage to the mammillary bodies occurs in infants with HIE irrespective of normothermia or hypothermia management.^10^, ^11^ This damage results in mammillary body atrophy that can be seen at 10 years of age in the absence of CP, and is associated with reduced hippocampal volume, altered white matter microstructure, and lower scores on neuropsychological tests than children with normal mammillary bodies following HIE.^12^ However, outcomes of those with and without mammillary body abnormalities have not been compared with typically developing controls. Additionally, it is not known how these mammillary body abnormalities are associated with the microstructure of the MTT and fornix.

In this study, we investigated the presence of mammillary body abnormalities in children aged 6 to 8 years who were treated with therapeutic hypothermia for HIE and did not have CP, in comparison with controls matched for age, sex, and socioeconomic status. We also investigated the association between mammillary body abnormalities and cognitive outcomes, hippocampal volumes, and diffusion properties of the MTT and fornix.

## METHOD

### Participants

This study was conducted at the Clinical Research and Imaging Centre (CRiCBristol), University of Bristol, UK, with approval from the North Bristol Research Ethics Committee (identification number 15/SW/0148). Participants' assent was ensured, and informed written consent was obtained from the parents of participants.

Cases were sequentially selected from the cohort of children who received therapeutic hypothermia between October 2007 and November 2012 under a standard protocol for perinatal asphyxia‐induced moderate to severe encephalopathy confirmed by amplitude‐integrated electroencephalography (aEEG) assessment^13^ as previously described.^5^ Cases were aged 6 to 8 years and did not have a diagnosis of CP at 2 years and at 6 to 8 years based on assessment of motor function and neurological examination. We excluded children who were cooled outside the standard criteria, born before 35 weeks' gestation, had an additional diagnosis, for example a metabolic disorder, or did not have English as their primary spoken language.

Controls were recruited from schools around Bristol. Controls were matched to cases using an individual matching approach, and we later confirmed that those included in the analysis were matched at the group level by ensuring there were no significant group differences in matching factors (age, sex, and socioeconomic status). We included children who were born at more than 35 weeks' gestation, had not had perinatal asphyxia with HIE, and spoke English as their primary spoken language.

Socioeconomic status was measured for each participant, according to their postcode at examination, using the index of multiple deprivation as defined for England by the UK Government (www.gov.uk/government/statistics/english‐indices‐of‐deprivation‐2019). Each postcode in England is assigned a number, on a scale of 1 to 10, indicating the decile within which the local area is ranked in the country, from most deprived (1) to least deprived (10).

Cases underwent neonatal magnetic resonance imaging (MRI), which was qualitatively assessed, by an experienced perinatal neurologist (FC), for the presence and extent of brain injury. This was scored, in the basal ganglia and thalami, white matter, and cortex (each on a score of 0–3), and the posterior limb of internal capsule (score 0–2), where a higher number indicates more severe injury.^13^, ^14^ These scans were not of sufficient resolution to allow assessment of mammillary bodies at this age.

### Cognitive assessment

Cognitive outcome was measured (by psychologists led by JT) using the Wechsler Intelligence Scale for Children, Fourth Edition,^15^ which assesses subscales of working memory, processing speed, verbal comprehension, and perceptual reasoning. These subscales are used to derive the full‐scale IQ.

### MRI acquisition

T1‐weighted MRI and diffusion‐weighted imaging (DWI) data were acquired as described in Spencer et al.^5^ A child‐friendly, detailed explanatory video was sent to the family before assessment day and presented again on the day of the scan together with the typical sounds in the MRI scanner. Children were scanned using a 3 T Siemens Magnetom Skyra (Munich, Germany) and a receive‐only 32‐channel head coil. Head movement was minimized using cushions, and a film of their choice was projected onto a screen visible through the mirror assembly of the head coil. A T1‐weighted volumetric scan was obtained with the magnetization‐prepared rapid acquisition gradient echo sequence using the following parameters: echo time 2.19 ms; inversion time 800 ms; repetition time 1500 ms; flip angle 9°; field of view 234 mm × 250 mm; 176 slices; 1.0 mm isotropic voxels; generalized autocalibrating partially parallel acquisitions acceleration factor 4.^16^ DWI data were acquired with a multiband echo‐planar imaging sequence, using the following parameters: echo time 70 ms; repetition time 3150 ms; field of view 192 mm × 192 mm; 60 slices; 2.0 mm isotropic voxels, flip angle 90°, phase encoding in the anterior–posterior direction, in‐plane acceleration factor 2 (generalized autocalibrating partially parallel acquisitions),^16^ through‐plane multiband factor 2.^17^, ^18^, ^19^ Two sets of diffusion‐weighted images, each with *b* = 1000s mm^−2^ in 60 diffusion directions and an additional eight interspersed *b* = 0 images, were acquired with a blip‐up blip‐down sequence (giving 136 volumes).

### Mammillary body assessment

**FIGURE 1:**
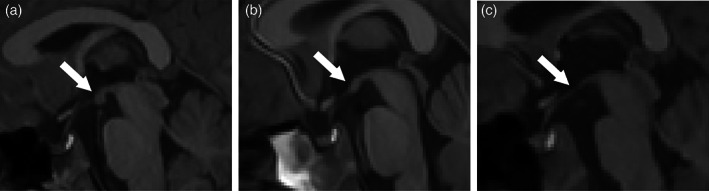
Mammillary body scoring on the sagittal plane of T1‐weighted images. Examples are given of (a) normal (score = 0), (b) equivocal (score = 1), and (c) abnormal (score = 2) mammillary bodies, indicated by the arrow in each panel.

The mammillary bodies were visually assessed on the sagittal plane of the T1‐weighted image by an experienced neonatologist (LSdV) and an experienced paediatric neuroradiologist (ML), both blinded to clinical status. Mammillary bodies were categorized as normal if there was no atrophy, equivocal if they appeared smaller than expected but were still visible, or abnormal if they were atrophic (appearing completely flat on sagittal T1‐weighted image) (Figure 1). Participants were rejected from the analysis if the mammillary bodies could not be scored because of artefacts on the T1‐weighted image.

### Hippocampal volumes

Volumes of the hippocampi were estimated from each individual's T1‐weighted scan using FIRST,^20^ the automated subcortical segmentation tool from the FMRIB software library (FSL, https://fsl.fmrib.ox.ac.uk/fsl).^21^ Visual inspection of hippocampal masks produced by FIRST, revealed that no manual correction was necessary.

We have previously reported case–control differences in whole‐brain volumes of both grey matter and white matter in this cohort.^7^ Therefore we normalized hippocampal volumes by total brain volume (TBV), which was calculated as follows. A brain tissue mask was created for each participant's T1‐weighted data using either SPM8‐VBM (http://fil.ion.ucl.ac.uk/spm)^22^ or the CAT12 module in SPM12 (http://www.neuro.uni‐jena.de/cat)^23^ depending on which gave better delineation of the brain surface. Subsequently, the brain was segmented into grey matter, white matter, and cerebrospinal fluid using FSL's FAST^24^ to obtain the whole‐brain volume of each tissue type. TBV was then calculated as the sum of grey and white matter volumes.

For this analysis, the T1‐weighted image had to be of sufficient quality to allow automated delineation of grey and white matter, and of the hippocampi. Therefore, the quality of T1‐weighted images was checked by assessors blinded to case–control status (JB and AS) and scans with excessive movement artefact were excluded.

### MTT and fornix microstructure

Microstructural properties of the MTT and fornix were measured for each individual by creating a group mask of each tract and measuring the diffusion properties within the mask from the co‐registered DWI data from each participant.

First, DWI data were corrected for eddy‐current‐induced distortions and participants' movements using Eddy^25^ and Topup^26^ from FSL. The quality of the DWI data was then assessed using FSL's EddyQC tool,^27^ and scans were rejected if the root‐mean‐square of movement and eddy current metrics was greater than one standard deviation above the mean for the whole cohort.

**FIGURE 2:**
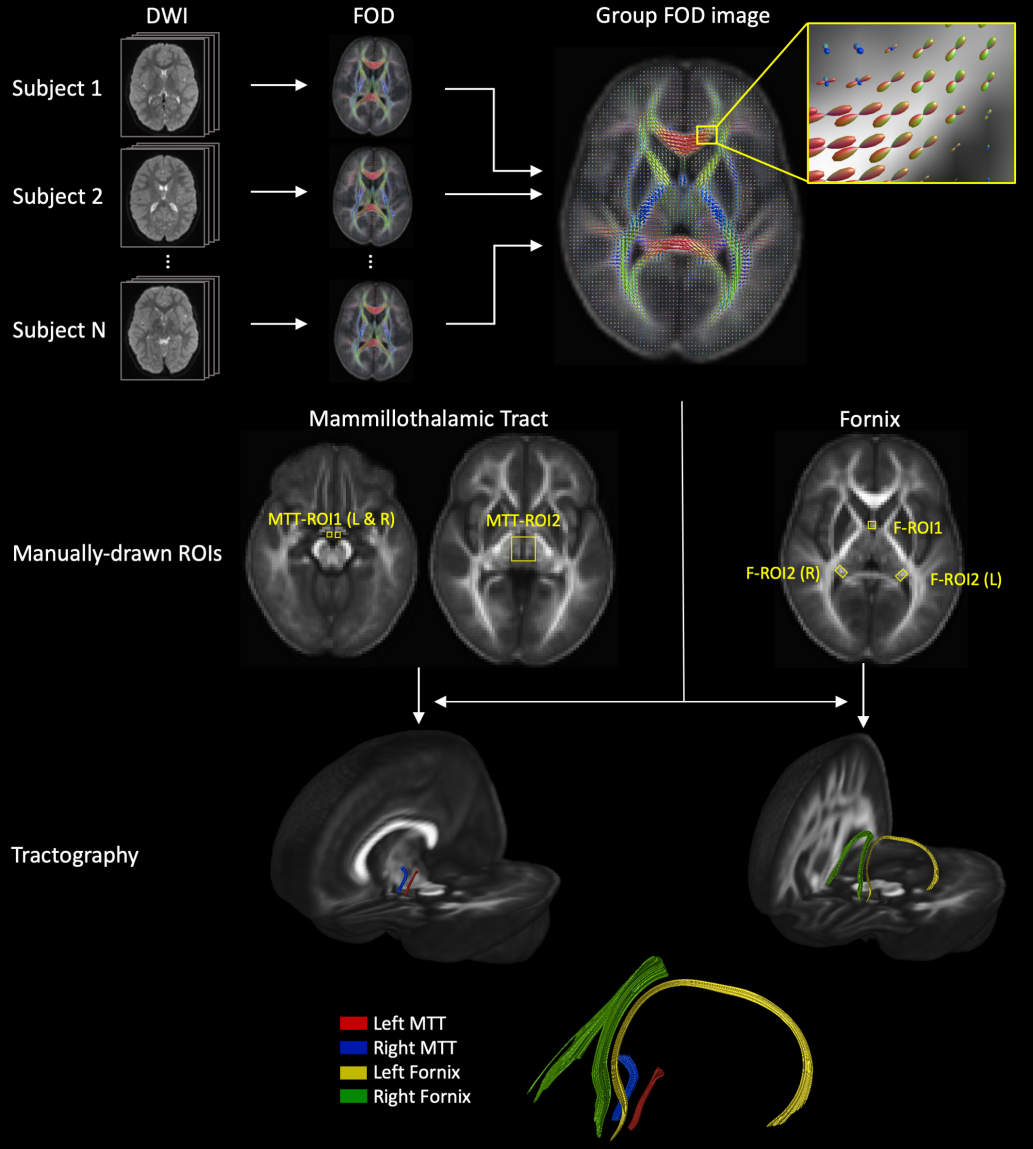
Method for delineating the left and right mammillothalamic tract (MTT) and fornix at the group level. Fibre orientation distribution (FOD) images were generated for all participants and combined to form a group FOD image, which was used to perform deterministic tractography. The zoomed‐in box shows the distribution of diffusion directions within individual voxels, indicating the distribution of underlying fibre orientations, with colour denoting the direction (red, green, and blue denote the *x*‐, *y*‐, and *z*‐axis respectively). Streamlines were generated from seed regions of interest (MTT‐ROI1 and F‐ROI1 for the MTT and fornix respectively) and included if they passed through target regions of interest (MTT‐ROI2 and F‐ROI2).

Subsequent DWI processing and tractography steps were performed using MRtrix 3.0 (www.mrtrix.org).^28^ The DWI signal for a typical fibre population (the response function) was estimated from the data^29^ and used to calculate the fibre orientation distribution (FOD) for each participant by deconvolving the response function from the measured DWI signal using constrained‐spherical deconvolution.^30^ A group FOD template was then created from all participants using the ‘population_template’ tool from MRtrix (Figure 2).

Deterministic tractography was performed using the group FOD image with the ‘SD Stream’ algorithm.^31^ Streamlines were generated with a step size of 0.2 mm and terminated if the angle between successive steps exceeded 60° or if the FOD amplitude dropped below 0.1 for the MTT or 0.2 for the fornix.

The left and right MTTs were delineated using the method described by Kamali et al.,^32^ as follows. A seed region of interest (ROI) was drawn over the mammillary body in the axial plane (MTT‐ROI1, Figure 2), then 1000 streamlines per voxel were seeded randomly within this ROI. A target ROI was drawn, in the axial plane at the level of the anterior commissure, around the streamlines generated in the region of the anterior thalamic nuclei (MTT‐ROI2, Figure 2). An AND operation was used to accept only streamlines that originated from the seed ROI and passed through the target ROI. Streamlines were truncated at the target ROI to isolate the MTT. This process was used to delineate the left and right MTTs by drawing seed ROIs over the left and right mammillary bodies respectively.

The fornix was delineated by placing a seed ROI around the columnar parts of the body (F‐ROI1, Figure 2), and a target ROI was placed around a unilateral crus (F‐ROI2, Figure 2).^33^, ^34^ The left and right fornices were delineated by placing target ROIs around the left and right crura respectively, with the same seed ROI.

A binary mask was then created for each tract by labelling any voxels that contained streamlines. To measure the tract‐level diffusion properties in each individual, fractional anisotropy and radial diffusivity images were generated for each participant by fitting a tensor model to the DWI data using the weighted least‐squares method in FSL's FDT software. These were subsequently transformed to common space using the transformations generated when creating the group FOD template described above. Each participant's fractional anisotropy and radial diffusivity in the left and right MTT and the left and right fornix were calculated by averaging values within the respective mask.

### Statistical analysis

Participants' demographics were compared between cases and controls using Mann–Whitney *U* tests or Fisher's exact tests. The case–control difference in mammillary body assessment scores (normal/equivocal/abnormal) was assessed using a Mann–Whitney *U* test, and the case–control difference in the number of participants with abnormal mammillary bodies was tested using a Fisher's exact test.

There were no differences in developmental outcomes of controls with normal mammillary bodies and controls with equivocal mammillary bodies (see Table S1); therefore the normal and equivocal groups were merged for subsequent analyses, giving three groups: controls; cases with normal or equivocal mammillary bodies; and cases with abnormal mammillary bodies.

We used a one‐way analysis of variance (ANOVA) to investigate differences between these three groups in cognitive outcome and hippocampal volume. For cognitive outcome, a separate test was performed for each domain of the Wechsler Intelligence Scale for Children, Fourth Edition (working memory, processing speed, verbal comprehension, perceptual reasoning, full‐scale IQ) then Bonferroni correction was applied for these five comparisons. For relative hippocampal volume, separate tests were performed for the left and right hippocampi then Bonferroni correction was applied for two comparisons. Post hoc two‐tailed *t*‐tests were used to assess pairwise group differences.

We then used a one‐way analysis of covariance (ANCOVA) to investigate group differences in diffusion properties of the MTT and fornix. As age and sex have both been shown to affect diffusion properties,^35^ these were included as covariates in this analysis. For the MTT, four tests were performed to assess both fractional anisotropy and radial diffusivity in both the left and right MTT, then Bonferroni correction was applied. Similarly for the fornix, four tests were performed to assess both fractional anisotropy and radial diffusivity in both the left and right fornix, then Bonferroni correction was applied. Post hoc two‐tailed *t*‐tests were used to assess pairwise group differences.

All *p*‐values reported were Bonferroni‐corrected, and *p* < 0.05 was considered significant. We used SPSS version 26 (IBM Corp., Armonk, NY, USA) for statistical analysis.

## RESULTS

### Recruitment

Fifty cases and 43 controls were recruited. Seven cases and four controls did not want to undergo scanning. Eleven cases and four controls had T1‐weighted images that were not of sufficient quality for mammillary body assessment, leaving 32 cases and 35 controls suitable for assessment of mammillary body abnormalities and cognitive outcomes. Of these, one case and three controls had T1‐weighted images that were not of sufficient quality for volumetric analysis, leaving 31 cases and 32 controls for assessment of hippocampal volumes, while four cases and two controls had DWI data that were rejected, leaving 28 cases and 33 controls for assessment of diffusion properties. Participants' demographics are shown in Table 1. There were no significant differences between cases and controls in age, sex, or deprivation index.

**TABLE 1:** Participants' demographics and results of mammillary body assessment

	Case (*n* = 32)	Control (*n* = 35)	*p*
Median age (IQR), years:months	7:0 (6:7–7:7)	7:4 (6:7–7:7)	0.995
Sex, male, *n* (%)	15 (47)	18 (51)	0.808
Median deprivation index (IQR)	7 (4–9)	7 (6–9)	0.237
Assisted ventilation at 10 minutes of age, *n* (%)	25 (78)		
Cardiac compressions required, *n* (%)	10 (31)		
Median Apgar score (IQR) at 10 minutes of age	6 (4.25–7.00)		
Median worst pH (IQR) within 1 hour of birth	6.94 (6.83–7.08)		
aEEG abnormalities before therapeutic hypothermia, *n* (%)			
Moderate	29 (91)		
Severe	3 (9)		
Mammillary body score, *n* (%)			0.007*
Normal (0)	16 (50)	26 (74)	
Equivocal (1)	5 (16)	9 (26)	
Abnormal (2)	11 (34)	0 (0)	
Abnormal mammillary bodies, *n* (%)	11 (34)	0 (0)	<0.001*

Perinatal clinical information is shown for cases but was not available for controls. *p < 0.05.

Abbreviation: aEEG, amplitude‐integrated electroencephalography; IQR, interquartile range.

Anatomical images were visually assessed for focal lesions and abnormal signal intensities. Small, non‐specific white matter signal changes were seen in two controls and no cases. These were judged by a blinded assessor (FC) to be minor and the scans were therefore not excluded.

### Mammillary body abnormalities

Out of 32 cases, 16 had normal mammillary bodies, five were equivocal, and 11 were abnormal (Table 1). Out of 35 controls, 26 had normal mammillary bodies, nine equivocal, and none were abnormal. Overall, the mammillary body scores were higher across the case group than controls (*p* = 0.007) and the difference in abnormal mammillary bodies between cases and controls was highly significant (*p* < 0.001). There was no difference between cases with and without mammillary body abnormalities in the proportion requiring assisted ventilation at 10 minutes of age, the proportion requiring cardiac compressions, Apgar score at 10 minutes of age, worst pH within 1 hour of birth, or the proportion with moderate/severe aEEG abnormalities before therapeutic hypothermia (Table 2). Asymmetry in mammillary bodies was noted in one case child with equivocal mammillary bodies, one control child with equivocal mammillary bodies, and one control child with normal mammillary bodies. No asymmetry was seen in any children with abnormal mammillary bodies.

**TABLE 2:** Comparison of perinatal clinical characteristics between cases with abnormal mammillary bodies and those with normal or equivocal mammillary bodies

	Cases with abnormal mammillary bodies (*n* = 11)	Cases with normal or equivocal mammillary bodies (*n* = 21)	*p*
Assisted ventilation at 10 minutes of age, *n* (%)	7 (64)	18 (86)	0.197
Cardiac compressions required, *n* (%)	5 (45)	5 (24)	0.252
Median Apgar score (IQR) at 10 minutes of age	5 (1–8)	6 (5–7)	0.659
Median worst pH (IQR) within 1 hour of birth	6.90 (6.80–6.98)	6.94 (6.83–7.11)	0.499
aEEG abnormalities before therapeutic hypothermia, *n* (%)			0.266
Moderate	9 (82)	20 (95)	
Severe	2 (18)	1 (5)	

Abbreviation: aEEG, amplitude‐integrated electroencephalography.

Scores of qualitative assessment of neonatal MRI were comparable between cases with and without abnormal mammillary bodies for white matter, the posterior limb of internal capsule, and basal ganglia and thalami. Cortex injury scores were higher for cases with abnormal mammillary bodies (*p* = 0.038).

### Cognitive outcomes

**FIGURE 3:**
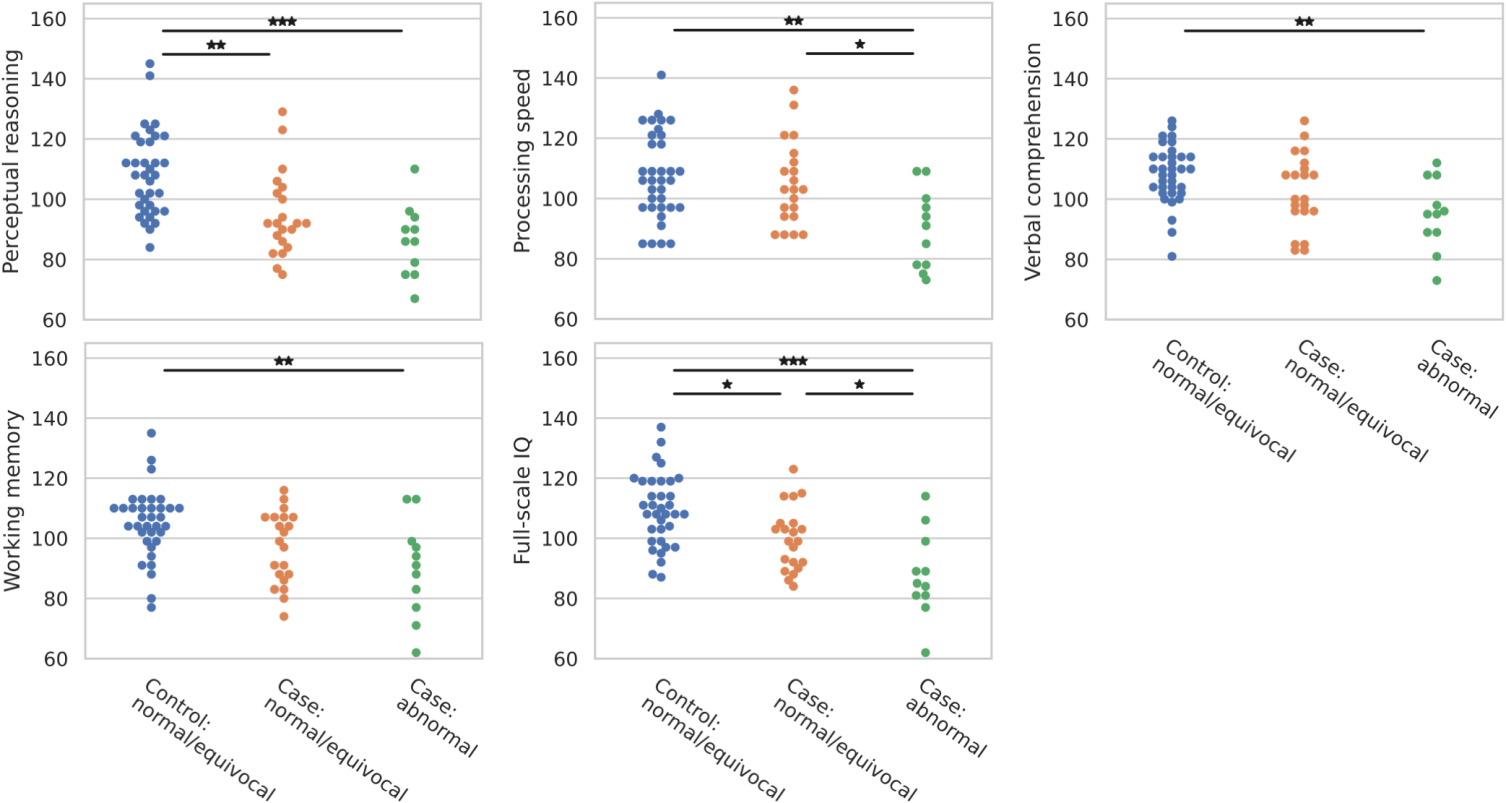
Comparison of cognitive scores between cases with abnormal mammillary bodies, cases with normal or equivocal mammillary bodies, and controls (all of which had normal or equivocal mammillary bodies). The significance of Bonferroni‐corrected post hoc *t*‐tests is indicated by *p < 0.05, **p < 0.01, ***p < 0.001.

When comparing cases with abnormal mammillary bodies, cases with normal or equivocal mammillary bodies, and controls, there were group differences (one‐way ANOVA) in all domains of the cognitive assessment: perceptual reasoning (*p* < 0.001); processing speed (*p* = 0.012); verbal comprehension (*p* = 0.011); working memory (*p* = 0.008); and full‐scale IQ (*p* < 0.001) (Figure 3). Post hoc tests revealed that cases with abnormal mammillary bodies had lower scores than controls in all domains: perceptual reasoning (*p* < 0.001); processing speed (*p* = 0.002); verbal comprehension (*p* = 0.002); working memory (*p* = 0.002); and full‐scale IQ (*p* < 0.001). Cases with normal or equivocal mammillary bodies had lower scores than controls for perceptual reasoning (*p* = 0.002) and full‐scale IQ (*p* = 0.015). Cases with abnormal mammillary bodies had lower scores than cases with normal or equivocal mammillary bodies in processing speed (*p* = 0.016) and full‐scale IQ (*p* = 0.028).

### Hippocampal volumes

**FIGURE 4:**
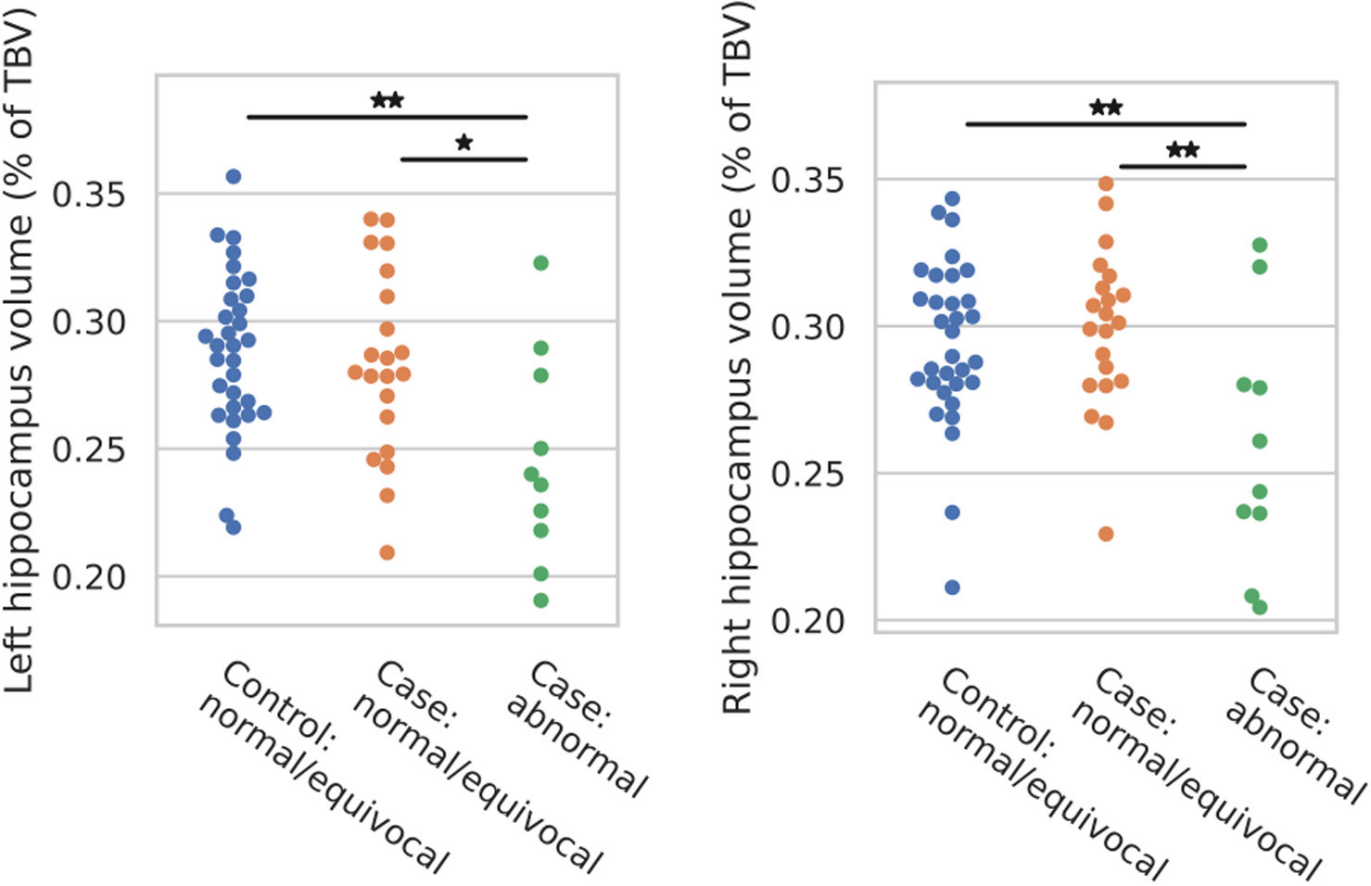
Comparison of hippocampal volumes, as a percentage of total brain volume (TBV), between cases with abnormal mammillary bodies, cases with normal or equivocal mammillary bodies, and controls (all of which had normal or equivocal mammillary bodies). The significance of Bonferroni‐corrected post hoc *t*‐tests is indicated by *p < 0.05, **p < 0.01, and ***p < 0.001.

There were group differences (one‐way ANOVA) in the volumes of the left (*p* = 0.008) and right (*p* = 0.007) hippocampi as a fraction of TBV (Figure 4). Post hoc tests revealed that relative hippocampal volumes in cases with abnormal mammillary bodies were smaller than in cases with normal or equivocal mammillary bodies (left *p* = 0.016; right *p* = 0.004), and smaller than in controls (left *p* = 0.003; right *p* = 0.008). There were no differences between cases with normal or equivocal mammillary bodies and controls.

### MTT microstructure

**FIGURE 5:**
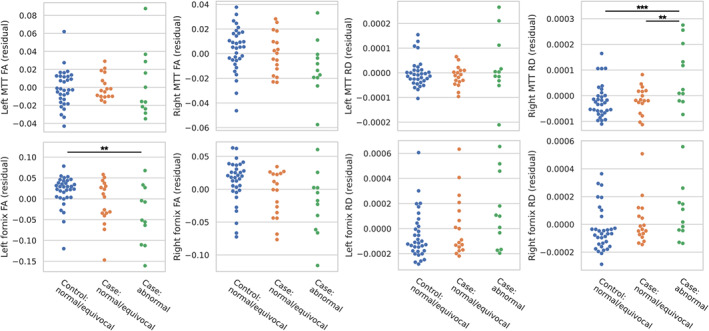
Comparison of fractional anisotropy (FA) and radial diffusivity (RD), in the left and right mammillothalamic tract (MTT) and fornix, between cases with abnormal mammillary bodies, cases with normal or equivocal mammillary bodies, and controls (all of which had normal or equivocal mammillary bodies). Metrics were compared using analysis of covariance, with age and sex included as covariates. Plots show residual values after regressing age and sex. The significance of Bonferroni‐corrected post hoc *t*‐tests is indicated by *p < 0.05, **p < 0.01, and ***p < 0.001.

There were group differences in the radial diffusivity in the right MTT (*p* = 0.003, one‐way ANCOVA; Figure 5). Post hoc tests revealed that radial diffusivity in the right MTT in cases with abnormal mammillary bodies was higher than cases with normal or equivocal mammillary bodies (*p* = 0.004) and higher than controls (*p* < 0.001). Radial diffusivity did not differ between cases with normal or equivocal mammillary bodies and controls. There were no group differences in the fractional anisotropy in the left and right MTT, or radial diffusivity in the left MTT. Including socioeconomic status as a covariate in this analysis had no impact on the results.

### Fornix microstructure

There were group differences in the fractional anisotropy in the left fornix (*p* = 0.017, one‐way ANCOVA; Figure 5). Post hoc tests revealed lower fractional anisotropy in the left fornix in cases with abnormal mammillary bodies than controls (*p* = 0.004). There were no group differences in the radial diffusivity in the left and right fornix, or fractional anisotropy in the right fornix. Including socioeconomic status as a covariate in this analysis had no impact on the results.

## DISCUSSION

In this study, we have shown that early school‐age children treated with therapeutic hypothermia for HIE at birth and who did not develop CP were more likely to have abnormalities of the mammillary bodies seen on MRI at 6 to 8 years compared with matched controls. When comparing outcomes between three groups – cases with abnormal mammillary bodies, cases with normal or equivocal mammillary body, and controls – there were group differences in all domains of the cognitive assessment. Additionally, there were group differences in the volume of the left and right hippocampi as a fraction of TBV, radial diffusivity in the right MTT, and fractional anisotropy in the left fornix.

In neonates with HIE, Molavi et al.^10^ reported that 11% of those cooled for HIE had mammillary body abnormalities, which is probably a conservative estimate due to large slice thickness. Lequin et al.^11^ later reported mammillary body abnormalities in 41% of cooled neonates. Annink et al.^12^ studied mammillary bodies in children aged 10 years with HIE; of those treated with therapeutic hypothermia (none with CP), 50% had abnormal mammillary bodies. In our cohort of children aged 6 to 8 years treated with therapeutic hypothermia without CP, 34% had abnormal mammillary bodies. We found no mammillary body abnormalities in a matched cohort of typically developing controls.

In a cohort with and without therapeutic hypothermia, Annink et al.^12^ reported that mammillary body abnormalities were associated with lower performance IQ, verbal IQ, and total IQ scores, as well as lower verbal long‐term memory and visual–spatial long‐term memory scores. We found that processing speed and full‐scale IQ were reduced in cases with abnormal mammillary bodies compared with those with normal or equivocal mammillary bodies, and all domains of the Wechsler Intelligence Scale for Children, Fourth Edition assessment were reduced in cases with abnormal mammillary bodies compared with controls. We found no differences in processing speed, verbal comprehension, or working memory between cases with normal or equivocal mammillary bodies and controls. This suggests that previously reported case–control differences in these domains from this cohort^3^, ^5^ are largely driven by the subgroup of cases with abnormal mammillary bodies who have reduced cognitive scores.

In the aforementioned study by Annink et al.,^12^ children with abnormal mammillary bodies were found to have smaller hippocampi as a percentage of TBV than those with normal mammillary bodies. Another study of 9‐ to 10‐year‐old children, not treated with therapeutic hypothermia, demonstrated reduced hippocampal volumes, as a percentage of intracranial volume, in those with moderate HIE compared with controls.^36^ We recently reported reduced hippocampal volumes in children treated with therapeutic hypothermia compared with controls; however, this was not independent of TBV.^7^ In the current study on the same cohort, we found that case children with abnormal mammillary bodies had reduced hippocampal volumes as a proportion of TBV, compared with case children with normal or equivocal mammillary bodies and compared with controls, while hippocampal volumes in cases with normal or equivocal mammillary bodies were comparable to those in controls.

The mammillary bodies contribute to memory function via their projections to the anterior thalamic nuclei through the MTT.^8^, ^9^ Alterations to the MTT have been associated with memory function in patients with Korsakoff syndrome,^9^ and associated with cognitive function in patients with subarachnoid haemorrhage.^37^, ^38^ We found that radial diffusivity in the right MTT was increased, indicating altered microstructure in these fibres, in cases with abnormal mammillary bodies compared with cases with normal or equivocal mammillary bodies and compared with controls. The significant alterations in radial diffusivity in the right MTT, and in fractional anisotropy in the left fornix, were not significant in the opposing hemisphere, but the same trends were exhibited. Additionally, no asymmetry was observed in the abnormal mammillary bodies; therefore this laterality may simply be due to the small sample size, and the limited specificity resulting from average diffusion metrics within each tract, rather than an underlying difference between hemispheres.

The mammillary bodies and the hippocampus reciprocally impact each other: the hippocampus projects to the mammillary bodies via the fornix,^8^ and the mammillary bodies are thought to play a role in regulating hippocampal–cortical oscillations via their projections to the anterior thalamic nuclei (through the MTT).^39^, ^40^ We found that the right MTT was altered in cases with abnormal mammillary bodies, compared with cases with normal or equivocal mammillary bodies and with controls, similar to our hippocampal findings. This may be due to a causal link between mammillary body abnormalities and reduced hippocampal volumes. Previous studies found that 42% to 50% of neonates with mammillary body abnormalities following HIE seemed to have no other brain abnormalities, suggesting the mammillary bodies are a primary site of injury.^10^, ^11^ These studies also reported that 20% to 33% had abnormalities in both the mammillary bodies and the hippocampi and suggest that, given the proximity of the assessment to the time of injury, it is likely that these are both primary sites of injury. This is also supported by the fact that some of those with abnormal mammillary bodies seemed to have hippocampal volumes comparable to the control group (Figure 4).

This is the first study, to our knowledge, to include a matched control group when assessing mammillary body abnormalities and associated cognitive outcomes in children with HIE. Case–control comparisons of hippocampal volumes and cognitive outcomes have been previously reported in this cohort.^5^, ^7^ This work analysed hippocampal volumes and cognitive scores in association with mammillary body abnormalities, and provided novel findings of altered diffusion properties in the MTT and fornix. The main limitation was the small sample size, which may have resulted in the false negatives, particularly in the investigation of diffusion properties of the MTT and fornix. For fibre tracking, we used a high angular resolution acquisition combined with constrained‐spherical deconvolution, followed by group‐level tractography to eliminate spurious between‐individual differences in the quality of delineation of these tracts. A possible limitation of this approach was the loss of specificity to each participant.

## CONCLUSION

We identified an increased occurrence of mammillary body abnormalities in early school‐age children treated with therapeutic hypothermia for HIE without CP compared with matched, typically developing controls. The subgroup of cases with mammillary body abnormalities had reduced cognitive scores, smaller hippocampi, and altered MTT and fornix microstructure compared with cases without abnormal mammillary bodies, and controls.

## Funding information

The Baily Thomas Charitable Fund (TRUST/VC/AC/SG4681‐7596), David Telling Charitable Trust, Sparks (05/BTL/01 and 14/BTL/01), and the Moulton Foundation. AS is supported by the Wellcome Trust (WT220070/Z/20/Z). JCWB is supported by the UK Medical Research Council (MR/N026969/1).

## CONFLICT OF INTEREST

The authors have stated that they had no interests that might be perceived as posing a conflict or bias.

## Supporting information


**Table S1:** Comparison of cognitive outcomes in controls with normal mammillary bodies to controls with equivocal mammillary bodies.

**Table S2:** Comparison of qualitative scores of injury on neonatal MRI between cases with abnormal mammillary bodies and cases with normal or equivocal mammillary bodies.

**Table S3:** Results from ANCOVA of radial diffusivity in the right mammillothalamic tract.

**Table S4:** Results from ANCOVA of fractional anisotropy in the left fornix.

## Data Availability

The data that support the findings of this study are available from the corresponding author upon reasonable request.
