# Supplementary material for: Mammillary body abnormalities and cognitive outcomes in children cooled for neonatal encephalopathy
Source: Dev Med Child Neurol. 2022 Nov 6;65(6):792–802. doi: 10.1111/dmcn.15453 (PMC10952753; doi:10.1111/dmcn.15453)
Supplement: Supplementary file 1 — Table S1: Comparison of cognitive outcomes in controls with normal mammillary bodies to controls with equivocal mammillary bodies. [file DMCN-65-792-s002.docx]

| Cognitive Domain | Controls with equivocal MBs (n=9) | Controls with normal MBs (n=26) | p |
| --- | --- | --- | --- |
| Perceptual Reasoning | 108 (84–125) | 108 (90–145) | 0.9246 |
| Processing Speed | 106 (91–128) | 106 (85–141) | 0.9094 |
| Verbal Comprehension | 112 (99–121) | 108 (81–126) | 0.5569 |
| Working Memory | 110 (97–126) | 104 (77–135) | 0.1646 |
| Full-scale IQ | 108 (92–127) | 109 (87–137) | 0.7476 |

Supplementary Table 1: Comparison of cognitive outcomes, displayed as median (range), in controls with normal MBs to controls with equivocal MBs. Scores did not differ between these two groups, so the normal and equivocal MB groups were merged in subsequent analyses.
