# Supplementary material for: Mammillary body abnormalities and cognitive outcomes in children cooled for neonatal encephalopathy
Source: Dev Med Child Neurol. 2022 Nov 6;65(6):792–802. doi: 10.1111/dmcn.15453 (PMC10952753; doi:10.1111/dmcn.15453)
Supplement: Supplementary file 2 — Table S2: Comparison of qualitative scores of injury on neonatal MRI between cases with abnormal mammillary bodies and cases with normal or equivocal mammillary bodies. [file DMCN-65-792-s001.docx]

|  | Cases with abnormal MBs (n=11) | Cases with normal or equivocal MBs (n=21) | p |
| --- | --- | --- | --- |
| White matter | 2 (0–3) | 1 (0–3) | 0.6164 |
| PLIC | 0 (0–1) | 0 (0–1) | 1.0 |
| BGT | 0 (0–1) | 0 (0–2) | 0.9276 |
| Cortex | 1 (0–3) | 0 (0–3) | 0.0380* |

Supplementary Table 2: Comparison of qualitative scores of injury on neonatal MRI between cases with abnormal MBs and cases with normal or equivocal MBs, for white matter, posterior limb of the internal capsule (PLIC), basal ganglia and thalamus (BGT), and cortex. Scores are shown as median (range), and Bonferroni-corrected p-values from Mann-Whitney U-tests are shown. *p<0.05.
