# Supplementary material for: Mammillary body abnormalities and cognitive outcomes in children cooled for neonatal encephalopathy
Source: Dev Med Child Neurol. 2022 Nov 6;65(6):792–802. doi: 10.1111/dmcn.15453 (PMC10952753; doi:10.1111/dmcn.15453)
Supplement: Supplementary file 3 — Table S3: Results from ANCOVA of radial diffusivity in the right mammillothalamic tract. [file DMCN-65-792-s003.docx]

|  | Adjusted Right MTT RD | Standard Error | 95% CI |
| --- | --- | --- | --- |
| Cases with abnormal MBs | 906 x10^-6^ | 23 x10^-6^ | 859 x10^-6^, 952 x10^-6^ |
| Cases with normal/equivocal MBs | 804 x10^-6^ | 19 x10^-6^ | 767 x10^-6^, 841 x10^-6^ |
| Controls | 802 x10^-6^ | 13 x10^-6^ | 775 x10^-6^, 828 x10^-6^ |

Supplementary Table 3: Results from ANCOVA analysis of RD in the right mammillothalamic tract. Values shown are adjusted for covariates (age and sex).
