# Supplementary material for: Mammillary body abnormalities and cognitive outcomes in children cooled for neonatal encephalopathy
Source: Dev Med Child Neurol. 2022 Nov 6;65(6):792–802. doi: 10.1111/dmcn.15453 (PMC10952753; doi:10.1111/dmcn.15453)
Supplement: Supplementary file 4 — Table S4: Results from ANCOVA of fractional anisotropy in the left fornix. [file DMCN-65-792-s004.docx]

|  | Adjusted Left Fornix FA | Standard Error | 95% CI |
| --- | --- | --- | --- |
| Cases with abnormal MBs | 0.275 | 0.015 | 0.245, 0.305 |
| Cases with normal/equivocal MBs | 0.306 | 0.012 | 0.282, 0.331 |
| Controls | 0.334 | 0.009 | 0.316, 0.351 |

Supplementary Table 4: Results from ANCOVA analysis of FA in the left fornix. Values shown are adjusted for covariates (age and sex).
